# Results of the Second Life Metabolism Travel Awards 2024

**DOI:** 10.1093/lifemeta/loae007

**Published:** 2024-03-20

**Authors:** John R Speakman

**Affiliations:** Shenzhen Key Laboratory of Metabolic Health, Center for Energy Metabolism and Reproduction, Shenzhen Institute of Advanced Technology, Chinese Academy of Sciences, Shenzhen, Guangdong 518055, China; State Key Laboratory of Molecular Developmental Biology, Institute of Genetics and Developmental Biology, Chinese Academy of Sciences, Beijing 100101, China; Institute of Biological and Environmental Sciences, University of Aberdeen, Aberdeen, AB24 2TZ, Scotland, United Kingdom

This year is the second year of the Life Metabolism Travel Prize Awards for PhD students and postdocs. The three 700 US$ awards are generously sponsored by Sable Systems–Promethion (China) who manufacture metabolic chambers. Before I announce the winners and their interesting projects, I will just reiterate how the judging of the awards works. We first remove anything from the submission that would identify the sex, affiliation, or ethnicity of the applicant. The anonymized abstracts are then sent to a panel of six independent reviewers. Each reviewer gets six votes to allocate to the abstracts that they think should win. So the maximum anyone could get would be six votes. This year we had three submissions that each polled four votes each. These are our winners. In no particular order, they are Sebastian Kreimendahl from the Pernas lab at the University of California Los Angeles (UCLA), Dipsikha Biswas from the Sakamoto lab at the Novo-Nordisk Foundation Center for Basic Metabolic Research (CBMR), University of Copenhagen, and Xinyue Qi, from the Hui lab at Nanyang Technological University (NTU).

Congratulations to all our winners. We hope you enjoy the conferences you attend using this money. Commiserations to those of you who entered but were unsuccessful this year. Keep an eye out for the announcement of the 2025 competition. Sable Systems-Promethion (China) have just confirmed that they will sponsor more awards in 2025. Thanks to them again for their generous support. Here are the research interests of our winners for 2024 summarized in their own words.

## Sebastian Kreimendahl, UCLA, Lena Pernas lab

Research interest: function of mitochondria to defend against invading microbes.


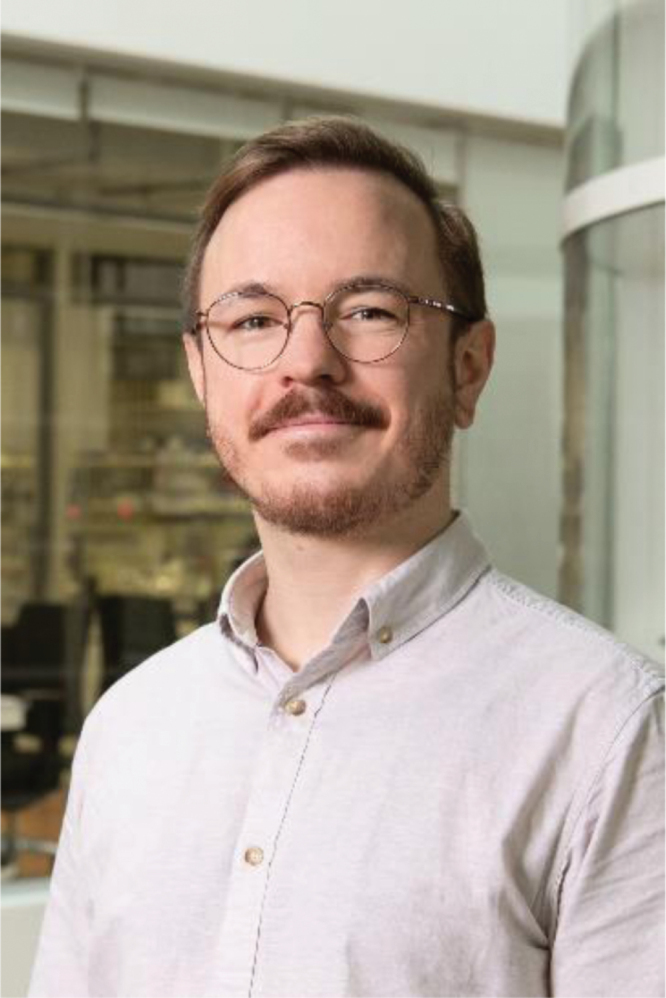


Mitochondria are critical immune signaling platforms in the host defense against intracellular pathogens. Consequently, mitochondria are a target of microbial effectors, several of which alter mitochondrial morphology and function independently of their role in innate immune signaling. This raises the question of whether mitochondria mediate noncanonical cell autonomous defenses during infection, for example by rewiring metabolic pathways to the detriment of the invader. In previous work, we found that host mitochondria compete with the human parasite *Toxoplasma gondii* for fatty acids, thereby restricting parasite growth. Using *Toxoplasma* as a model, I am currently exploring how an infected cell can weaponize mitochondria to defend against invading microbes. To address this question, I performed a mitochondria-specific loss-of-function CRISPR/Cas9 screen which revealed several mitochondrial pathways as restrictors or promotors of the intracellular growth of *Toxoplasma*. My results shed light on how mitochondria regulate microbial growth, and conversely, how *Toxoplasma* exploits mitochondrial function to its benefit.

## Dipsikha Biswas, University of Copenhagen, Kei Sakamoto lab

Research interest: nutrient signaling and metabolism in the muscle.


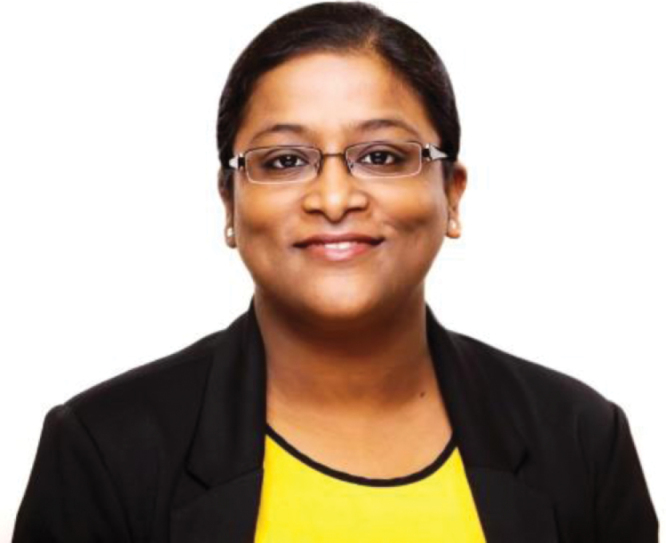


I am a senior postdoctoral fellow in the laboratory of Prof. Kei Sakamoto at CBMR, University of Copenhagen. My research career has revolved around nutrient signaling and metabolism in the muscle. In the Sakamoto lab, I got the opportunity to investigate a fundamental question about the physiological regulation of muscle glycogen phosphorylase (PYGM) and glycogen breakdown, a primary form of energy supply during exercise. PYGM is a critical glycogen-degrading enzyme during exercise or prolonged fasting. Loss of function mutation of PYGM causes a metabolic myopathy known as McArdle’s disease, characterized by severe exercise intolerance, premature fatigue, and muscle weakness during exercise. PYGM activity is regulated by binding of allosteric activator 5ʹ-adenosine monophosphate (AMP) and phosphorylation of its Ser15 residue induced by epinephrine. The relative role of these two molecular switch mechanisms in controlling muscle glycogen breakdown *in vivo* is unknown. We identified an AMP-insensitive PYGM mutant through structure-guided mutagenesis and generated AMP-insensitive (V46T) and phospho-deficient (S15A) PYGM knock-in (KI) mice. Both the KI mouse models were viable and mutant PYGM V46T and S15A protein abundance was unchanged. Robustly increased (~6-fold) glycogen content was observed in S15A muscles, while V46T muscles showed a modest increment (~1.4-fold). Specific loss of function mutation of PYGM (V46T or S15A) did not alter glucose homeostasis, energy expenditure, or metabolic phenotype of the mice. Loss of function in the KI mice was validated by evaluating enzyme activity *in vitro* and measuring biochemical parameters in response to AMP/AMP mimetic (AICAR) and epinephrine in isolated muscles. AMP/AICAR-induced reduction of glycogen *ex vivo* was attenuated while epinephrine-induced Ser15 phosphorylation/enzyme activation and reduction in glycogen content remained intact in the V46T muscles. In S15A muscles, AMP/AICAR, but not epinephrine, stimulated PYGM activity and glycogen degradation. For functional analysis, mice were subjected to *ex vivo* muscle contractions and treadmill running exercise, and strikingly, we observed that muscle glycogen breakdown required phospho-regulation of PYGM but AMP regulation was dispensable. Interestingly, despite minimal glycogen degradation in S15A mice, their voluntary running capacity was similar to WT mice. In summary, we generated and validated AMP-insensitive V46T and phospho-deficient S15A KI mouse models to dissect the role of AMP and phosphorylation for PYGM regulation and glycogen breakdown in the muscle during physical exercise.

## Xinyue Qi, NTU, Hui Ye lab

Research interest: cholesterol metabolism, with a focus on the function of 27-hydroxycholesterol (27HC).


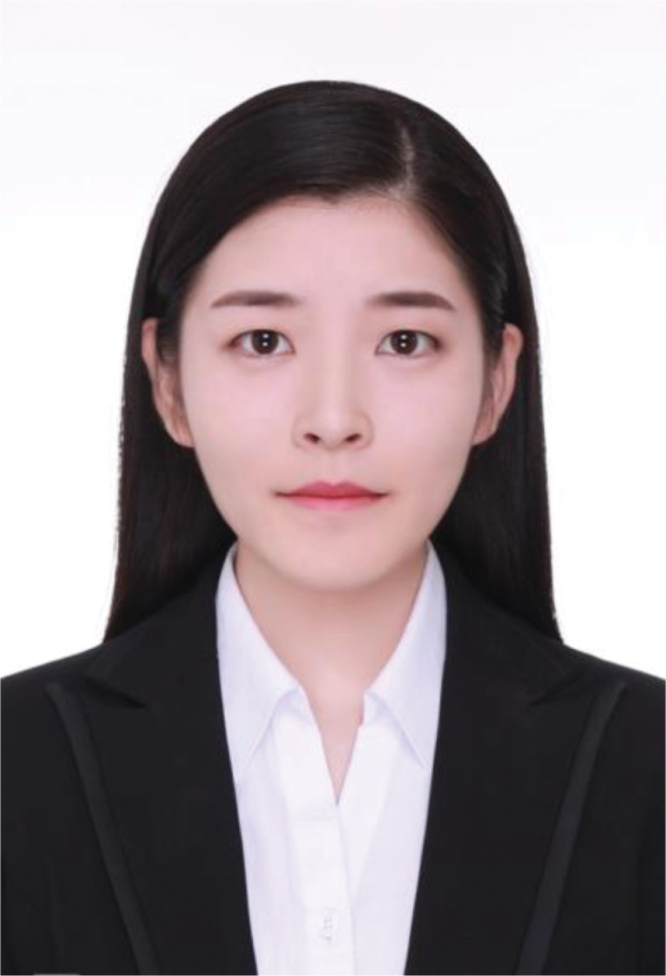


Oxysterols are metabolites of cholesterol produced in peripheral tissues as a means to eliminate cholesterol. 27HC is the most abundant oxysterol and can cross the blood-brain barrier. Interestingly, 27HC has recently been identified as an endogenous selective estrogen receptor modulator (SERM) for both estrogen receptor α and β (ERα/β). Considering the regulatory effects of brain estrogen/ERα signaling on energy metabolism, we hypothesize that the endogenous SERM 27HC binds with ERα in the arcuate nucleus of the hypothalamus (ARH) of the brain to modulate energy homeostasis. In supporting this point of view, we found that a single acute intracerebroventricular (ICV) injection of 27HC inhibited food intake in both male and female mice. The reduced food intake was attributed to decreased meal size and increased intermeal intervals. This anorexigenic effect was also associated with the increased c-fos expression in the pro-opiomelanocortin neurons in ARH (POMCARH). Using brain slice patch-clamp recording, we consistently showed that 27HC dose-dependently activated POMCARH in an ERα-dependent manner, suggesting a mediating role of ERα expressed by POMCARH neurons. Notably, we further revealed that the inhibitory effects of 27HC on food intake were blocked by antagonists for ERα or POMC downstream melanocortin 3/4 receptors. In addition, chemogenetic inhibition of POMCARH neurons and specific deletion of ERα in POMC also blunted the anorexigenic effects of 27HC in mice. Collectively, these results support a model that 27HC acutely inhibits food intake by acting on ERα to stimulate POMCARH neuronal activity. This 27HC/ERα/POMC signaling pathway may serve as a critical defense mechanism against high-fat diet-induced obesity.

